# Cluster analysis as an effective tool for identifying physical fitness in students: the basis for an innovative approach to optimizing physical education in the university environment

**DOI:** 10.3389/fphys.2025.1634125

**Published:** 2025-08-29

**Authors:** Alena Cepková, Richard Cepka, Ľubomír Šooš, Oto Honz, Marián Uvaček, Ján Žiška, Erika Zemková

**Affiliations:** ^1^ Institute of Languages, Physical Education and Social Sciences, Faculty of Mechanical Engineering, Slovak University of Technology in Bratislava, Bratislava, Slovakia; ^2^ Institute of Manufacturing Engineering and Quality Production, Faculty of Mechanical Engineering, Slovak University of Technology in Bratislava, Bratislava, Slovakia; ^3^ Department of Biological and Medical Sciences, Faculty of Physical Education and Sports, Comenius University in Bratislava, Bratislava, Slovakia

**Keywords:** endurance, flexibility, health risks, muscle strength, performance testing, speed

## Abstract

**Objective:**

This study analyzes university students’ physical fitness and, based on the results, applies cluster analysis to identify homogeneous groups with aim to optimize physical education programs at the university.

**Methods:**

A group of 88 first-year students underwent standardized UNIFITTES 6-60 focusing on strength (long jump from a place, sit-ups in 30 s, bent-arm hang test), endurance (20 m shuttle run test), speed (4 × 10 m shuttle run), flexibility (sit and reach test), and anthropometric measurements to determine BMI and WHR. Cluster analysis was used to identify homogeneous groups based on students’ physical fitness and anthropometric profiles.

**Results:**

The average BMI reached the value of 23.95, with 12% of students falling into obesity. An increased risk of cardiovascular diseases were identified in 19% (WHR). The distance in standing long jump was 212.3 ± 29.2 cm, the number of sit-ups in 30 s was 228.2 ± 4.3 repetitions, the time in bent-arm hang test was 44.9 ± 30.6 s, the reaching distance in the sit and reach test was 4.2 ± 8.8 cm, the time of the 4 × 10 m shuttle run test was 10.4 ± 0.7 s, the distance covered in the 20 m shuttle run test was 45.4 ± 18.6 runs, and the right and left hand grip strength was 50.8 ± 9.6 kg and 49.1 ± 8.7 kg, respectively. Using cluster analysis and ANOVA, three significantly different performance groups were identified: cluster 0 ≼ cluster 1 ≼ cluster 2.

**Conclusion:**

These findings indicate that cluster analysis is an effective tool for distinguishing physical fitness levels in students. Identification of their performance profiles allows for the optimization of physical education programs.

## 1 Introduction

Currently, there is seen a growing interest in the health and physical fitness of the young population. Special attention is being paid to the group of university students, who are often prone to a decrease in their physical activities and worsening of lifestyle due to study load, stress and changed lifestyle habits. Research consistently confirms the importance of regular physical activity for physical and mental health, quality of life, and academic performance (Castelli et al., 2007; Gil-Espinosa et al., 2020; Herbert, 2022; Al Doghan and Sadiq, 2024; Luzano, 2024; Teuber et al., 2024; Zayed et al., 2024). For example, students who participated in physical activity interventions significantly improved their math performance, while language and reading skills did not change significantly (Cipriano et al., 2024). Positive lifestyle changes, especially regular physical activity and healthy eating habits, contribute to higher levels of physical fitness and improved mental health (García-Pérez-de-Sevilla and Pérez-Chao, 2021; Herbert, 2022; Martín-Rodríguez et al., 2024). Greater physical fitness is also associated with enhanced cognitive performance, optimal brain development, and increased self-esteem (Gil-Espinosa et al., 2020; Castelli et al., 2007; Zayed et al., 2024). Specifically, exercise interventions involving low-to moderate intensity aerobic exercises improve mental health (alleviate depressive symptoms and perceived stress) among university students (Herbert, 2022). In contrast, low levels of physical activity are linked to increased health risks, including obesity, cardiovascular diseases, and musculoskeletal imbalances (Plotnikoff et al., 2015; Leuciuc, 2017; Ikenna et al., 2022; Latino et al., 2023). Oladejo et al. (2023) highlighted the high prevalence of overweight and obesity among students. Furthermore, he emphasized the importance of physical activity for optimizing BMI and WHR, which are key health indicators. Students unhealthy lifestyle behaviors, including low physical activity, increase the risk of overweight and obesity (Telleria-Aramburu and Arroyo-Izaga, 2022). In light of these findings, schools and universities represent key settings for promoting physical activity and assessing physical fitness as part of health promotion and disease prevention strategies (Pate et al., 2006; World Health Organization, 2018). In the case of university students, systematic reviews indicate that although their level of physical activity and physical fitness is generally satisfactory, there are differences resulting from cultural and educational factors (Kljajević et al., 2021). Supporting physical fitness assessment in this population is important, as college students often experience a decline in their physical activity as a result of academic workload and lifestyle changes (Kljajević et al., 2021). The increasing emphasis on improving healthy lifestyles and preventing chronic diseases at the university level stimulates the research focused on effective and personalized approaches in physical education.

Cluster analysis is one of the progressive methodological tools that finds its application in the field of sports and health sciences. This allows to identify homogeneous groups of individuals based on selected parameters and subsequently adapt interventions to meet their specific needs. The basic methodological works include the work by Everitt et al. (2001), which offers a comprehensive view of the principles and applications of clustering, as well as by Kaufman and Rousseeuw (1990), who provides a systematic overview of the methods for identifying groups based on data - with high potential for applications in sports sciences. In the field of applied research, interesting results are provided by studies in which physical fitness and anthropometric indicators in university students are analyzed (Hart, 2017). Cai et al. (2023) used the hierarchical cluster analysis to identify students with different fitness profiles and compared differences between groups based on the measures such as VO_2_max and body fat percentage. Similarly, Burns et al. (2017) validated a set of fitness tests (including measurements of grip strength, flexibility, and abdominal and hip flexor muscular endurance) through the cluster classification of students. Bennasar-Veny et al. (2020) found out clustering of risk factors such as smoking, poor diet and physical inactivity, thus indicating the need for a comprehensive approach. Murphy et al. (2019) confirms the existence of behavioral clusters in the university population, that were significantly associated with demographic factors such as gender and income level. El Ansari et al. (2018) highlight in their study conducted in England, Wales and Northern Ireland that behavioral profiles of students are not homogeneous that proves the need for personalization. This knowledge supports the importance of combining performance variables (20 m shuttle run, 4 × 10 m shuttle run, long jump, sit-ups, bent-arm hang, sit and reach) and anthropometric indicators (BMI, WHR, waist and hip circumference) in classifying students in order to target physical development.

These studies offer different perspectives for the assessment of physical fitness and its relationship to health risks in university students. However, despite the increasing use of cluster analysis in sports and health sciences, there is a noticeable lack of studies specifically targeting non-athlete male students from technical universities. Most existing research focuses on athletes or mixed student populations, leaving a gap in knowledge regarding this specific subgroup. Addressing this gap may improve the effectiveness of targeted health and physical education strategies in university settings. The application of cluster analysis in this area can provide a deeper understanding of different fitness profiles and their relationship to health indicators, which can be the basis for more effective interventions and recommendations.

This study analyzes university students’ physical fitness and, based on the results, applies cluster analysis to identify homogeneous groups with aim to optimize physical education programs at the university. We hypothesize that cluster analysis will identify three distinct groups of students with significantly different levels of physical fitness and body composition. This assumption is based on prior research using cluster analysis in university student populations (e.g., Burns et al., 2017; Cai et al., 2023; Bennasar-Veny et al., 2020), where three distinct fitness profiles—typically low, moderate, and high—frequently emerged. In addition, preliminary analysis of our data using silhouette coefficient and dendrogram inspection supported a three-cluster solution as the most interpretable and statistically robust.

## 2 Methods

### 2.1 Participants

A group of 88 male university students volunteered to participate in this study (Table 1). They attended the 1st to 3rd years of bachelor’s study programs at the Slovak University of Technology in Bratislava, in the academic year 2023/24. The study sample consisted of 88 male students from one university, which represents a limitation due to the small sample size and lack of female participants and broader geographical diversity. Therefore, the results should be interpreted with caution, and their generalization to the wider student population is limited. However, this study provides valuable pilot insights that can serve as a basis for future, larger-scale studies involving more diverse samples.

**TABLE 1:** Descriptive statistics of participants characteristics.

	Mean (SD)	Min	25%	Median	75%	Max
Age (yr)	20.1 (1.6)	18	19	20	20.3	25
Height (cm)	180.2 (6.9)	157.5	175.0	180.3	185.0	196.0
Body mass (kg)	77.9 (18.2)	55	66	73	83.3	151
Waist circumference (cm)	80.8 (13.5)	60	72	76	89.3	129
Hip circumference (cm)	95.8 (12.5)	64	87	95	103	135
BMI	24.0 (5.1)	15.9	20.7	22.4	25.3	43.7
WHR	0.84 (0.06)	0.66	0.79	0.85	0.89	0.99

SD, standard deviation; Min, minimum value; 25%, first quartile; 75%, third quartile; Median, median; Max, maximum value.

Inclusion criteria: The absence of regular sports activity, participants were not involved in the organized regular sports training or did not perform intensive physical activity more than three times a week. The absence of serious health problems, students had to safely complete the physical performance tests.

Exclusion criteria: Serious musculoskeletal diseases that would make the participation in measurements impossible. Elite athletes with regular training loads were excluded from testing to avoid distortion and misrepresentation of physical performance indicators. Urgent and serious health problems at the time of testing that might affect current physical performance.

This research was in accordance with the ethical standards on human experimentation stated in compliance with the 1964 Helsinki Declaration and its subsequent modifications. This project was approved by the ethics committee of the Faculty of Physical Education and Sports, Comenius University in Bratislava (No. 2/2023). All experimental procedures were clearly explained to the participants, who then provided a written consent before the commencement of testing. Informed consent was obtained from all participants included in the study.

### 2.2 Design

All students were tested during the first physical education lesson. Initially, students were informed about the testing procedure and completed an initial screening. In the second stage, anthropometric tests were performed: body height (cm), body weight (kg), waist circumference (cm) and hip circumference (cm). Body Mass Index (BMI) was subsequently calculated by the equation BMI weight (kg)/height (m)^2^ and Waist-to-Hip Ratio (WHR) = waist circumference (cm)/hip circumference (cm). Waist and hip circumference (measured with a tape measure while standing, at the widest points). Subsequently, standardized physical performance tests were performed according to UNIFITTES 6-60: shuttle run 4 × 10 m (speed, agility), long jump from a place (explosive strength of the lower limbs), sit-ups in 30 s (dynamic strength of the abdominal and hip-thigh muscles), the bent-arm hang test (strength of the upper limbs), 20-m shuttle run test – 20MSR (aerobic endurance), hand dynamometry (grip strength of the dominant and non-dominant hand), sitting forward bend with reach (flexibility). The UNIFITTEST 6–60 is a standardized battery of physical fitness tests developed for assessing individuals aged 6–60 years. It includes a range of motor tests designed to evaluate strength, endurance, flexibility, and speed (Měkota and Kovář, 1995).

We focused on anthropometric and physical fitness tests because the primary aim was to objectively assess physical fitness profiles and identify related health risks in university students. These tests provide standardized, quantifiable data directly related to physical condition and performance, which are essential for tailoring physical education programs. Although dietary habits and mental health are important factors influencing overall health and fitness, they were beyond the scope of this study due to design and resource limitations. Future research should include these aspects for a more comprehensive understanding.

### 2.3 Statistical analysis

For subsequent analysis, we aggregated the raw measurements into five composite scores representing key physical fitness dimensions. Prior to aggregation, all individual variables were normalized to the range between 0 and 1 using Min-Max scaling. This ensured that each variable contributed equally to the composite scores, preventing variables with larger numerical ranges from dominating the results. For variables where a lower value indicates better performance (specifically, 4 × 10 m shuttle run, BMI, and WHR), the normalized score was inverted by subtracting it from 1 so that higher scores consistently represent better performance across all metrics. The normalized variables were then combined into five specific fitness scores, and the total performance score as follows:

• Strength (St): An average of the normalized scores for DNM RH (kg), DNM LH (kg), long jump from the place (cm), sit-ups in 30 s (reps), and bent-arm hang (s). This composite score integrates multiple aspects of muscular strength from different body parts (upper limbs, lower limbs, and the core), reflecting the multidimensional nature of strength as established in physical fitness literature (Měkota and Kovář, 1995).
•Endurance (En): The normalized score for 20-m shuttle run test (number of runs).
•Speed (Sp): The inverted normalized score for 4 × 10 m shuttle run (lower time is better).
•Flexibility (Fl): The normalized score for sit and reach (cm).
•Proportions (Pr): An average of the inverted normalized scores for BMI and WHR (lower values are considered better).

Finally, a Fitness index (Fi) was calculated as the average of these five composite scores.

Prior to conducting the statistical tests, we checked the required assumptions (normality and equal variance). While ANOVA and independent samples t-tests are generally robust to moderate violations of normality, we formally tested normality using the Shapiro-Wilk test at a significance level of 0.05 (if not mentioned otherwise). However, the equal variance was tested using Bartlett’s test, and if the test failed, we would proceed with Welch’s t-test instead of the standard independent samples t-test. Multiple simultaneous tests were adjusted by Bonferroni correction.

## 3 Results

### 3.1 Health and physical fitness characteristics of university students

The BMI of 23.95 ± 5.09 (Table 1) is within the normal range according to the classical BMI categorization (18.5–24.9), which indicates that the majority of students (62.5% of students) are generally not at significant risk related to obesity or underweight. For students (17%), whose BMI values are above 25, above the upper limit of the normal range, it indicates a higher risk of developing metabolic diseases, such as the type 2 diabetes, cardiovascular problems, and an increased risk of high blood pressure. Possible health risks may be indicated within very high BMI values, above 30 (12% of students) are measured. In the future, in these students health problems may occur, such as obesity, diabetes, heart disease, and musculoskeletal diseases. The average measured WHR value of 0.84 ± 0.06 (Table 1) indicates a normal range, without health problems. WHR ≧0.9 (19% of students) indicates a higher risk of obesity, cardiovascular diseases and metabolic disorders. The waist circumference was 80.87 ± 13.45 cm (Table 1), the values above 94 cm (9% of students) indicate an increased risk of the development of cardiovascular diseases and metabolic problems. The risk values of the waist circumference above 100 cm (9% of students) are considered as a signal for potential health problems, such as insulin resistance and the increased risk of heart diseases.

The physical fitness tests (Table 2) show that the average values indicate a medium to slightly above average level of physical performance in the sample studied (n = 88). Nevertheless, there is considerable variability within individual tests, which is also confirmed by the differences between the minimum and maximum values. In the long jump test from a place of 212.33 ± 29.21 cm (min 135 cm, max 276 cm), the difference of up to 141 cm between the weakest and the best performance indicates significant differences in the explosive power of the lower limbs. Students with values below 193.75 cm show weaker explosive power, while those with the value higher than 236 cm achieve above-average results. In the sit-ups test done in 30 s, there was 22.82 ± 4.26 repetitions (min 12, max 34), the performance below 20 repetitions indicates a weaker level of abdominal muscles, while the value above 25 repetitions (75%) indicates a better developed trunk muscle strength. In the bent-arm hang test (44.85 ± 30.64 s) some students were unable to maintain hold on the flexion for even a second (min = 0 s), while the best performance was 111 s. Such a large range reflects the different levels of static strength and the endurance of the upper limbs. In the flexibility test of sit and reach in a sitting position, the students achieved 4.16 ± 8.80 cm (min −12 cm, max 20 cm), the values below zero mean that the student is unable to reach the level of the toes in the forward bend. The range of 32 cm (from −12 cm to 20 cm) indicates different levels of flexibility in the hamstrings and lumbar part. In the 4 × 10 m shuttle run, students achieved the average performance of 10.44 ± 0.70 s (min 9.15 s, max 12.35 s). Results below 10.11 s indicate above-average speed abilities, while times above 10.65 s indicate weaker speed abilities.

**TABLE 2:** Descriptive statistics of study variables of physical fitness.

	Mean (SD)	Min	25%	Median	75%	Max
JUMP FROM A PLACE (cm)	212.33 (29.21)	135.00	193.75	212.00	236.00	276.00
SIT-UPS 30s	22.82 (4.26)	12.00	20.00	22.00	25.25	34.00
BENT-ARM HANG (s)	44.85 (30.64)	0.00	22.50	44.50	65.00	111.00
SIT AND REACH (cm)	4.16 (8.80)	−12.00	−2.50	3.50	12.00	20.00
RUN 4 × 10 (s)	10.44 (0.70)	9.15	10.11	10.34	10.64	12.35
20MSR (count)	45.35 (18.63)	10.00	32.75	45.50	60.00	100.00
DNM RH (kg)	50.78 (9.63)	21.30	44.78	50.80	58.00	72.20
DNM LH (kg)	49.09 (8.73)	21.90	44.68	49.80	54.12	69.40

Endurance abilities were tested with the use the 20-m shuttle run test. Students achieved 45.35 ± 18.63 runs, (min 10, max 100). The difference of up to 90 runs between the weakest and strongest performance indicates a large difference in endurance abilities in students. In the right hand dynamometry test (DNM RH), students achieved 50.78 ± 9.63 kg (min 21.3 kg, max 72.2 kg). Higher values above 58 kg mean higher grip strength. On the contrary, the values below 44.78 kg indicate a weak grip. When testing the left hand, the average values in dynamometry (DNM LH) were lower 49.09 ± 8.73 kg (min 21.9 kg, max 69.4 kg). The range is similar to that of the right hand, but on average slightly lower values (1–2 kg) indicate the frequent right-sided dominance in most right-handed people.

### 3.2 Physical fitness clustering analysis

**FIGURE 1:**
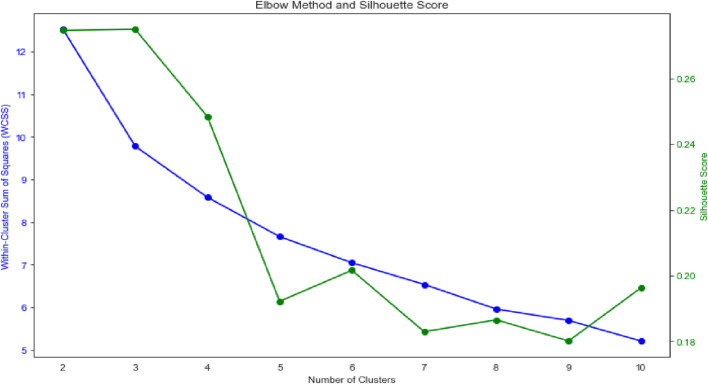
Elbow method and silhouette score.

We performed K-means cluster analysis on our dataset. Based on the Silhouette Score and the Within-Cluster Sum of Squares (WCSS) elbow plot, we concluded that the data contains three weakly separated clusters (Figure 1). The WCSS measures the total squared distance between each point and its assigned cluster centroid. The idea is to find a point (the “elbow”) where adding more clusters does not significantly decrease the WCSS anymore. The Silhouette Score measures how well-separated clusters are. It considers both the cohesion (how close points are within their own cluster) and separation (how far points are from neighbouring clusters). Scores range from −1 to 1, with higher values indicating better-defined clusters.

Based on a cluster analysis conducted on five base composite scores (St, En, Sp, Fl, Pr), we hypothesized the existence of three distinct performance groups (clusters) within our data (Figure 1), ordered by performance as follows: cluster 0 ≼ cluster 1 ≼ cluster 2. Consequently, we aimed to statistically test the hypothesis that the mean scores for Fitness index follow this order: μ_Fi0_ < μ_Fi1_ < μ_Fi2_. Our testing strategy involved two steps: first, we performed a one-way ANOVA to test for overall differences among the group means (μ_Fi0_, μ_Fi1_, μ_Fi2_), which yielded a significant result (p < 0.001). Subsequently, we conducted two *post hoc* independent samples t-tests to confirm the specific pairwise differences μ_Fi0_ < μ_Fi1_ (p-value <0.001, Cohen’s d = 2.04) and μ_Fi1_ < μ_Fi2_ (p-value <0.001, Cohen’s d = 1.80), normality for the Fi2 was assessed at the 0.01 significance level.

Therefore, we concluded that the observed differences among the three performance groups are statistically significant, supporting our hypothesis based on the cluster analysis.

**FIGURE 2:**
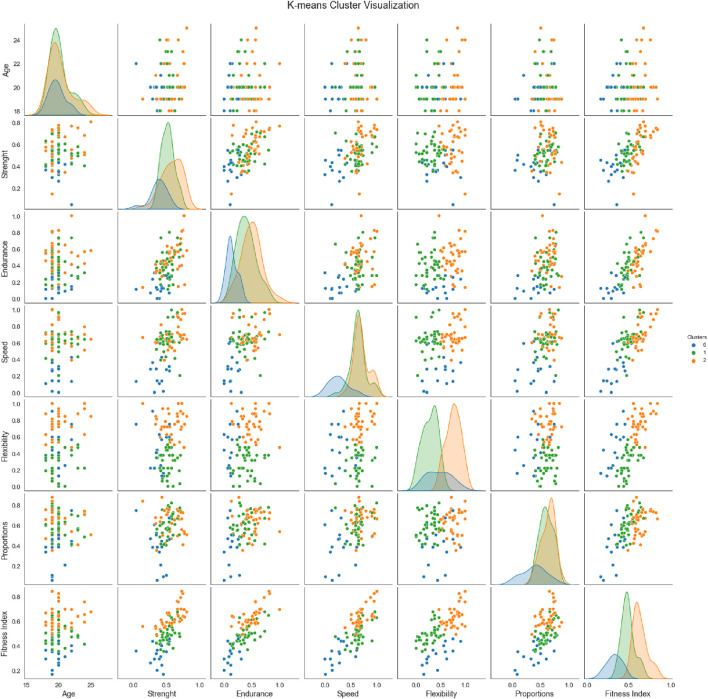
K-means cluster visualization.

Cluster 0 (blue group) has the lowest average fitness index (Figure 2). From the cluster analysis, we found out that this group is significantly behind in the strength (μ_St0_ < μ_St1_, p-value <0.001), endurance (μ_En0_ < μ_En1_, p-value <0.001 Welch’s t-test) and speed components (μ_Sp0_ < μ_Sp1_, p-value <0.001), normality for the Sp1 was assessed at the 0.01 significance level. Based on the statistical analysis, recommended training interventions should include the basic general fitness development, i.e., balanced additional strength exercises with lower intensity to support speed and power, and introduction of aerobic activities (cycling, swimming, walking) to improve endurance. This group may suffer from a higher risk of overweight and metabolic diseases (e.g., diabetes, hypertension). Low muscle mass can lead to a back and joint pain, while poor fitness can result in a faster start of fatigue and a higher resting heart rate. With regard to motivation, it would be suitable to include, for example, team sports (Table 3).

**TABLE 3:** Physical education teaching strategies based on clusters.

Cluster	Focus	Recommendations	Example exercises
Cluster 0	Basics, motivation	To increase the basic fitness skills, develop endurance and strength	Bodyweight squats, modified push-ups, brisk walking, swimming, light resistance band exercises, team sports like soccer or basketball for motivation
Cluster 1	Balanced development	To develop the specific physical capabilities aimed at reducing weak performance, and to maintain balanced development	Steady jogging, dynamic leg swings, lunges with torso twist, yoga-based stretching, cycling
Cluster 2	Performance training	Performance support, challenges, leadership of others	Olympic lifts, medicine ball throws, resistance sprints, plyometrics (box jumps, jump squats), HIIT sessions

Cluster 1 (green group), according to the fitness index, this group is characterized as a group with average performance (Figure 2). It includes students with a good body composition (proportions, low BMI and WHR) but worse flexibility (μ_Fl1_ < μ_Fl2_, p-value <0.001). We can also conclude that they are behind the most trained students (cluster 2) in endurance (μ_En1_ < μ_En2_, p-value <0.05). Therefore, we recommend longer runs in the Z2 heart rate zone with dynamic stretching to build endurance and flexibility for this group (Table 3).

Cluster 2 (orange group), according to the Fitness index, this group is defined as the group with the best performance. Students in this group achieved the highest scores in flexibility, endurance and strength (Figure 2). These are the students with good strength performance and overall fitness, good explosive ability and range of motion. A higher proportion of muscle mass is assumed. Recommended training interventions include power-explosive exercises (e.g., olympic weightlifting, medicine ball, resistance sprints, plyometric exercises). High-intensity training is also suitable (Table 3).

These results suggest that personalized training programs can significantly contribute to the optimization of physical fitness development in students in the university-level physical education, as well as prevention of injuries.

## 4 Discussion

The results showed that although the average body composition values correspond to the normative ranges, a significant proportion of students show values that may represent increased health risks.

The average BMI value in the sample was 23.95 (62.5% students), which corresponds to the WHO classification of “normal weight” (range 18.5–24.9). However, 17% of students had the BMI higher than 25, which already falls into the overweight category, and up to 12% exceeded the obesity threshold (BMI >30). These values are consistent with the findings of Bennasar-Veny et al. (2020), who identified a similar distribution of overweight and obesity in the university student population. WHR is an equally important risk indicator, the value of which was 0.84 on average in the sample. For men, the values above 0.90 are considered risky, which was exceeded by 19% of students. This indicator is associated with an increased risk of visceral obesity and metabolic syndrome, which is also confirmed by the research studies carried out by Czyż et al. (2017) and Greene et al. (2011). Their research studies report that central obesity is strongly correlated with low physical activity and poor nutritional habits. Studies show that higher BMI values are associated with an increased risk of the development of metabolic disorders, such as insulin resistance, type 2 diabetes and cardiovascular diseases. Himes and Dietz (1994), Lee et al. (2012), and Razak et al. (2020) showed that high BMI values in adults are strongly associated with a higher risk of cardiovascular diseases and metabolic disorders (obesity, type 2 diabetes).

As regards the waist circumference, the average value in the sample was 80.78 cm, which is below the threshold of 94 cm. According to international criteria, this indicates an increased risk for cardiovascular diseases in men. However, 9% of students exceeded this threshold, where 9% of them showed the values above 100 cm, that is considered as a clear indicator of metabolic dysfunction and risk of insulin resistance. Similar conclusions were presented by Görner and Reineke (2020), who demonstrated an association between the waist circumference and reduced cardiovascular performance in female students.

Although the overall values may give the impression of a healthy population, a more detailed analysis indicates the existence of a subpopulation with a higher risk of health problems, which is consistent with the findings reported by Greene et al. (2011) on the existence of the “risk clusters” of behaviors in university students. These clusters include combinations of the factors such as lack of physical activity, unhealthy eating habits, and excessive body fat. Students who participated in physical activity for 2–3 h per week demonstrated a higher probability of academic success. The study conducted by Lipošek et al. (2019) included students from the University of Maribor aged 20–22 years. Physical fitness was evaluated using standardized test batteries Eurofit and FitnessGram. Academic success was operationally defined as regular progression to the second year of study. The achieved mean value of 212.33 cm is comparable to the results of Italian students, where men achieved an average of 211.58 cm after a fitness program (Lovecchio et al., 2012). Similarly, a study conducted in a group of Slovenian students reported the average performance of 225.9 cm, which indicates a slightly higher level of explosive power in this population (Lipošek et al., 2019). In the bent-arm hang test, we found the average time of 44.85 ± 30.64 s, which is higher than the results of Italian students, who achieved the average of 42.53 s after completing a fitness training program (Lovecchio et al., 2012). The students in the 4 × 10 m shuttle run test achieved the average value of 10.44 ± 0.70 s, which is comparable to the results of Romanian students, who achieved the average of 13.58 s before the fitness program and 12.52 s after completing it (Lazăr and Leuciuc, 2021). These results indicate that the sample has a better level of speed and agility.

The average value of 22.82 ± 4.26 repetitions in the sit-ups test is lower compared to Slovenian students, who achieved the average of 49.8 repetitions in 60 s, which would correspond to approximately 24.9 repetitions in 30 s (Lipošek et al., 2019). These results indicate a similar level of trunk muscle strength between both groups. In the same research study, students achieved the average of 22.6 cm, while in our study it was lower, 4.16 ± 8.80 cm. The average value of 45.35 ± 18.63 runs in the endurance 20MSR test in our study achieved lower results than Slovenian students, who achieved the average of 38.2 mL/kg/min, which would correspond to a higher number of runs. These data indicate a lower level of endurance abilities of our students. When comparing hand dynamometry, the average value of 50.78 ± 9.63 kg (right hand) in students from the Technical University is lower than the results of Romanian students, who achieved the average value of 64.68 kg before the fitness program and 61.47 kg after completing it Lazăr and Leuciuc (2021). These data indicate a lower level of grip strength in our sample of students.

The results, visualized in the pair plot (Figure 2) show the high efficiency of K-means clustering in the assessment of physical fitness in university students. The identification of three clusters allows not only to distinguish the performance and proportion profiles of students, but also to design targeted training strategies and strategies for teaching physical education at the university level (Table 3). According to Czyż et al. (2017), cluster analysis provides valuable information for planning movement interventions based on physical abilities, body composition (BMI, WHR) and behavior.

Despite of overlap between clusters in some dimensions, several axes show clear separation (Figure 2), which is also commonly presented in foreign studies. Bennasar-Veny et al. (2020) demonstrated that university students cluster into homogeneous subgroups according to physical fitness, although some transitional features exist between them.

Our analysis shows that age is not a decisive factor in separating groups, this finding corresponds to the research results demonstrated by Bennasar-Veny et al. (2020), according to which chronological age is less decisive than performance or health indicators. Ortega et al. (2008) also confirmed that performance differences are not primarily influenced by age, but rather by the level of physical activity and motion competence. Based on the statistical analysis and the characteristics of the identified clusters, we recommend the implementation of personalized physical education interventions. For example, students in the group with the lowest performance may benefit from programs focused on gradual development of aerobic endurance and basic strength, while higher-performing students may respond better to specialized strength or mobility training. This differentiation may help improve motivation, health outcomes, and long-term exercise adherence.

This strategy is supported by research conducted by Greene et al. (2011) and Wackerhage and Schoenfeld (2021), who applied classification methods to design interventions for students with different health and performance profiles. Song and Ahn (2014) emphasize the importance of multivariate analyses (such as clustering) in designing programs that respect individual needs, not only from the perspective of physical fitness, but also health prevention.

The results show that three relatively distinct performance profiles can be identified in the student population, which can be used for targeted education and personalized movement interventions. This approach supports current trends in personalized physical education and training (Light and Kentel, 2013). Such an approach could lead to more effective development of motor skills in the context of university physical education. Ortega et al. (2008) focused on testing of cardiorespiratory fitness, muscle strength and speed/agility, where they confirmed that physical fitness should be considered as an important indicator of health. The results of cluster analysis can thus contribute to the identification of specific groups with different needs and to a more effective personalization of the approach to physical development.

This study has some limitations that may affect the interpretation and generalizability of the results. The inclusion criteria required that participants were not involved in organized regular sports training or did not perform intensive physical activity more than three times per week. This intentionally targeted a sample of students with lower levels of physical activity, which may limit the applicability of the findings to more active or elite athlete populations. The exclusion criteria involved removing elite athletes with regular training loads to avoid distortion and misrepresentation of physical performance indicators. Additionally, students with serious musculoskeletal diseases or acute health issues that could prevent safe participation in the tests were excluded.

These limitations allowed us to focus on the target group but at the same time narrowed the scope of generalization of the results to the broader population of university students, especially those who are physically active or regularly engaged in sports. Furthermore, due to organizational constraints, the study was conducted as a cross-sectional analysis without longitudinal follow-up. The absence of longitudinal data limits our ability to assess the stability of the identified clusters over time. Future studies should aim to incorporate repeated measurements to validate whether cluster membership remains consistent or changes as students progress through their studies or modify their physical activity behavior. In addition, unmeasured confounding factors such as psychological stress, sleep quality, or dietary habits may have influenced physical performance outcomes. These variables were not controlled for in the present study but should be considered in future research to better isolate the effects of physical activity and fitness.

## 5 Conclusion

Cluster analysis revealed three statistically different performance clusters. The combination of five base composite scores (strength, speed, endurance, flexibility, proportions) with physical development indicators (BMI, WHR) provides a valuable foundation for identifying groups with different physical fitness profiles. However, given the study’s limitations, these findings should be considered preliminary and require further validation in larger and more diverse populations, ideally with longitudinal data. Accordingly, these insights can contribute to designing more effective and attractive training programs, which are important for the long-term sustainability of physical activity and the prevention of health complications in the university student population.

## Data Availability

The raw data supporting the conclusions of this article will be made available by the authors, without undue reservation.
