# Supplementary figures and images for: Cluster analysis as an effective tool for identifying physical fitness in students: the basis for an innovative approach to optimizing physical education in the university environment
Source: Front Physiol. 2025 Aug 29;16:1634125. doi: 10.3389/fphys.2025.1634125 (PMC12425939; doi:10.3389/fphys.2025.1634125)

Table 1 histogram:

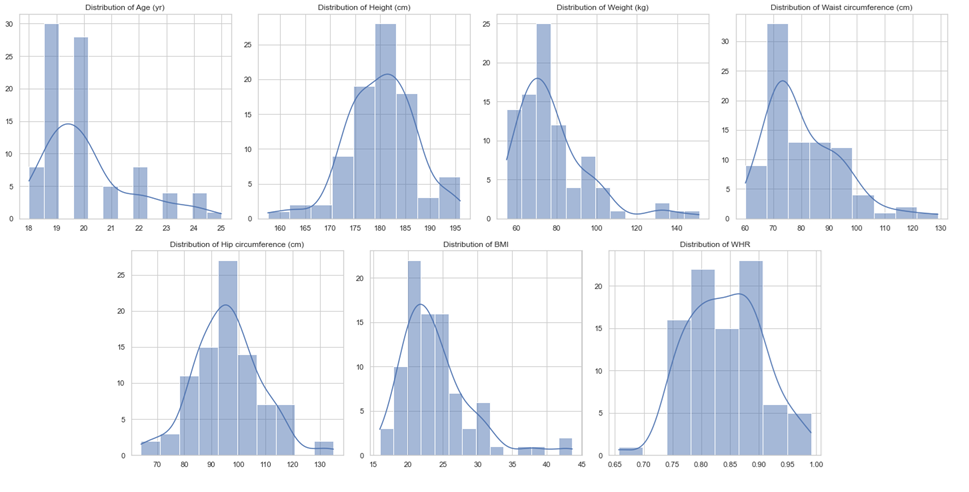


Table 2 histogram:
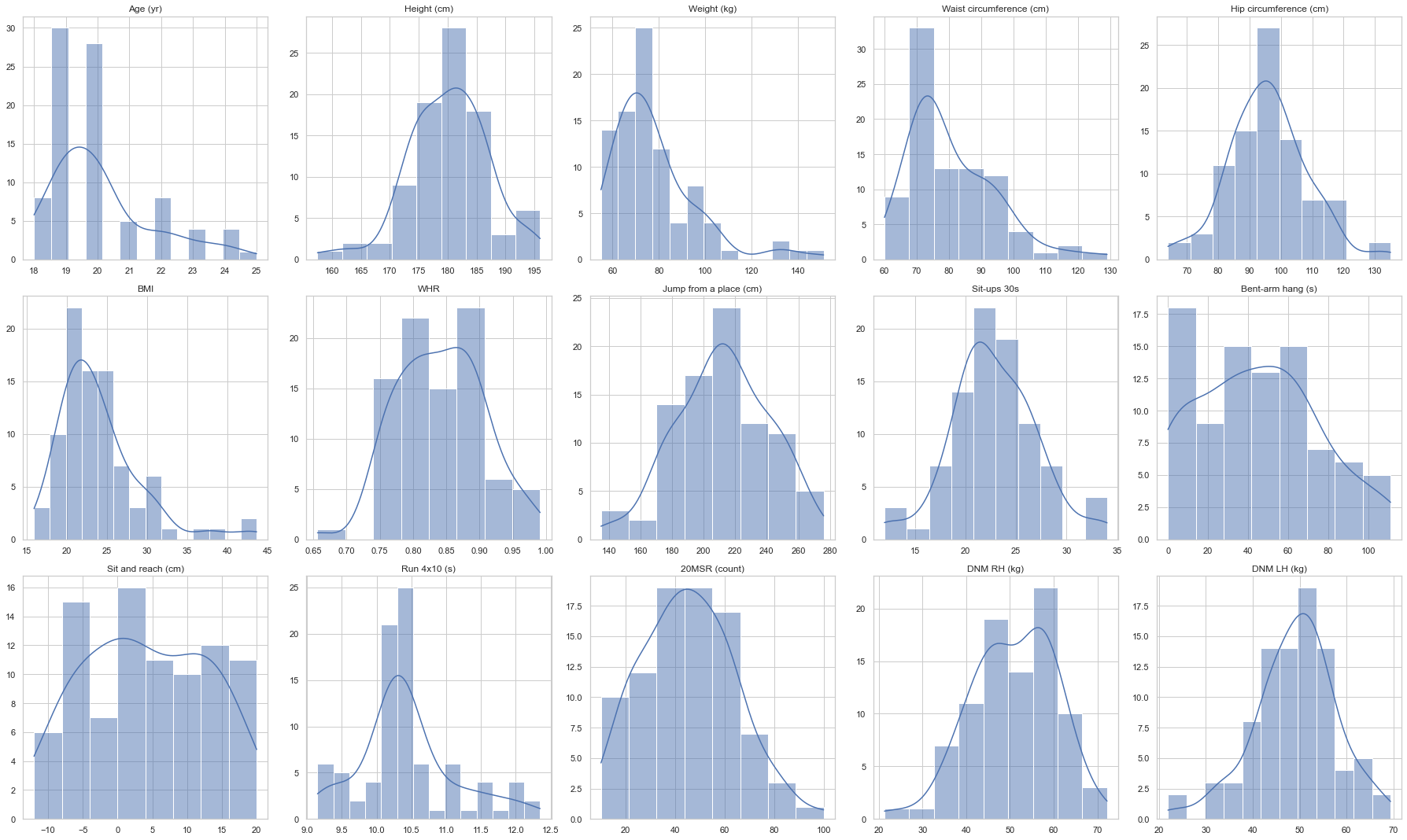

Supplement: Supplementary file 1 [file Table1.docx]
